# Effects of DTL electrode position on the amplitude and implicit time of the electroretinogram

**DOI:** 10.1007/s10633-019-09733-3

**Published:** 2019-11-04

**Authors:** Anna H. Brouwer, Gerard C. de Wit, Joke H. de Boer, Maria M. van Genderen

**Affiliations:** 1 Bartiméus Diagnostic Centre for Complex Visual Disorders, Zeist, The Netherlands; 2 Department of Ophthalmology, University Medical Centre Utrecht, Heidelberglaan 100, 3584 CX Utrecht, The Netherlands

**Keywords:** Electroretinogram (ERG), Electrophysiology, Dawson, Trick, and Litzkow (DTL) electrodes

## Abstract

**Purpose:**

This study sought to investigate whether there is an optimal position of the Dawson, Trick, and Litzkow (DTL) electrodes when measuring the full-field electroretinogram (ERG) for monitoring purposes.

**Methods:**

In 200 uveitis patients, an extended light-adapted (LA) ERG protocol was measured twice, incorporating the International Society for Clinical Electrophysiology of Vision standards. First, a LA ERG was measured with the DTL in the lower lid position (LLP) and thereafter in the fornix position. Differences in amplitudes and implicit times of a-waves, b-waves, and the 30 Hz peak were investigated. Intraclass correlation coefficients (ICCs) as well as coefficients of variation (CoV) were calculated, to assess both reliability and relative variability between the two DTL positions.

**Results:**

Implicit times showed no statistically significant differences between the two DTL positions. As expected, amplitudes at the different stimulus strengths were 1.12–1.19 higher in the LLP, but there were no significant differences in the CoV between the two DTL positions. The ICC was high for the b-wave and 30 Hz flicker response (0.842–0.979), but lower for the a-wave, especially for amplitudes (0.584–0.716).

**Conclusions:**

For monitoring purposes in patients, we conclude that based on relative variability, no position is preferable above the other. However, because in most diseases amplitudes are decreased, the LLP may be chosen because it yields higher amplitudes. Whatever the choice, it is important to ensure that the DTL position remains stable during an ERG recording.

**Electronic supplementary material:**

The online version of this article (10.1007/s10633-019-09733-3) contains supplementary material, which is available to authorized users.

## Introduction

Since the introduction of the Dawson, Trick, and Litzkow (DTL) electrode [1], its use in recording electroretinograms (ERGs) has spread. One of the main advantages of the DTL is that it is much more comfortable to wear compared to conventional electrodes, such as contact lens electrodes [2–4].

Originally, the ERG was mainly used for diagnosing retinal diseases such as retinal dystrophies, where the ERG is frequently severely abnormal. However, now that the ERG is increasingly used for monitoring disease, more subtle ERG changes become important. Therefore, one must be aware of factors that may affect the ERG results, other than disease or treatment. Particularly, factors that influence the inter-session variability are important.

Factors that may influence the absolute ERG results, but will have little effect on intersession variability because they do not differ between sessions, include gender [5], refraction [6], and ocular pigmentation [7]. Other factors that can affect the intersession variability may be minimized by always incorporating International Society for Clinical Electrophysiology of Vision (ISCEV) standards. These include duration of dark and light adaptation, flash strengths, and pupil size [8]. Besides these, there are factors that also affect the intersession variability but are more difficult to address. These include DTL position [9–12], media opacities [13], and age [14–16].

When the DTL electrode was introduced, it was said that its position was “only a little disturbed by blinking” [1]. However, since then several reports state that the DTL position can shift, which may significantly affect the recorded amplitudes.

When the DTL is positioned on the cornea, the highest amplitudes can be recorded, which decrease as the DTL is positioned more toward the fornix [17]. Despite these differences in amplitudes, the latest ISCEV standard does not advice which DTL position should be used. The most common DTL positions are the lower lid position (LLP) and the fornix position (FP) [8].

In our clinic, reference values are based on ERG measurements recorded at the LLP. This position was advised by the manufacturer for two reasons. First, it would ensure high amplitudes, without much risk of scratching the cornea. Second, blinking would have little effect on the DTL position. In some cases however, we have seen the DTL shift toward the fornix during an ERG recording.

Some studies advise using the FP, because it would shift less easily and therefore lead to a more stable recording. The FP would yield lower amplitudes, but the ERG would be less variable [9–11]. However, these studies were conducted in relatively small groups of healthy volunteers. These healthy volunteers are probably often coworkers who know how to cooperate during an ERG because they are familiar with ERG procedures. Therefore, it is useful to see whether the same results can be obtained in a large cohort of patients, who may be scared or photophobic which makes the recording of an ERG more difficult. Also, a larger group can yield more reliable confidence intervals of differences between the two DTL positions.

The aim of this study was to determine the optimal DTL electrode position for monitoring purposes. We investigated whether one DTL position yields more stable ERG results than the other. If such a difference is found, this would imply that this DTL electrode position should be used for monitoring purposes. We compared the reliability as well as the relative variability of the results and investigated differences in amplitudes and implicit times.

## Methods

### Subjects

The subject population, consisting of 200 patients (355 uveitis eyes, 45 unaffected eyes) with a non-infectious uveitis, aged ≥ 18 years (median 53.4, IQR 39.2–63.7), has been previously described [18]. All patients were mentally competent and gave their written consent to participate. This study was conducted in compliance with the ethical principles of the Declaration of Helsinki. Ethical approval was requested and obtained from the Medical Ethical Research Committee of the University Medical Centre Utrecht.

### ERG measurement

All ERGs were measured according to the ISCEV standards [8]. An Espion E3 system with colordome stimulator (Diagnosys LLC, Cambridge, UK) was used for full-field flash stimulation. Eyes were anesthetized with oxybuprocaine 0.4%. Pupils were dilated with tropicamide 0.5%. Cup electrodes were used as ground and reference electrodes and placed on the forehead and on the temples. Impedances of reference and DTL electrodes were below 5 kΩ and below 10 kΩ for the ground electrode.

An extended ISCEV protocol was measured twice, consisting of stimulus strengths that increase with approximately 0.5 log units and range from 0.3 to 10.0 cds/m^2^ for the light-adapted ERG (LA), including a 30 Hz flicker response (LA, 3.0 cds/m^2^). For analyses, averages were used of two results, each consisting of five sweeps. In cases when sweeps with a trend were accepted during recording, these sweeps were toggled, or removed.

**Fig. 1:**
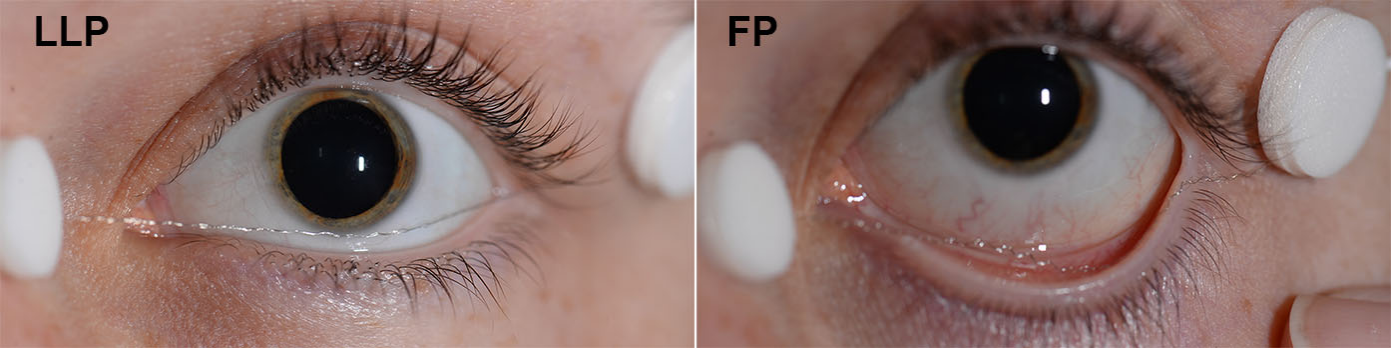
Example of the two DTL positions. Representative example of the two Dawson, Trick, and Litzkow (DTL) positions that were used: the lower lid position (LLP) (left) and the fornix position (FP) (right)

After 10 min of light adaptation, the first LA ERG was recorded with the DTL electrode in the LLP and the second with the FP (see Fig. 1). The DTL position was checked prior to each measurement and adjusted if necessary.

### Analyses

For statistical analysis, RStudio version 1.0.143 was used. To compare differences between the two DTL positions, a Wilcoxon signed rank test was used (data not normally distributed as tested with histograms, Q–Q plots and Shapiro–Wilk test).

We added a random value between − 0.25 and 0.25 ms to each implicit time, because our equipment measures every 0.5 ms. By adding this random value, the implicit time becomes a true continuous variable which has statistical advantages for calculating more reliable confidence intervals. The implicit time difference and amplitude ratio of the two DTL positions were calculated and plotted.

To evaluate the effect of the two DTL positions on the percentage of patients that would fall outside normal limits, we compared the results to our reference values. These reference values were obtained with the DTL in LLP and were previously described [18].

To investigate the amount of reliability between the two measurements, intraclass correlation coefficients (ICCs) were calculated (two-way model, type consistency, unit average). For visualization of the amount of agreement between the two positions, Bland–Altman plots were made as well.

To evaluate the amount of relative variability of the ERG data at the two DTL positions, we calculated the coefficients of variation (CoV, sd/mean), also known as relative standard deviation. The data were transformed (square root) to fit a normal distribution. This was necessary since the results of uveitis patients can range from normal to abnormal which gives a skewed distribution [18]. Normality was evaluated using Shapiro–Wilk tests, histograms and Q–Q plots. We used the R package cvequality (version 0.1.3; Marwick and Krishnamoorthy 2018) to test for significant differences in CoV. Using Bonferroni’s correction, we defined *P* values of < 0.006 as statistically significant. All significances were two-tailed.

## Results

**Fig. 2:**
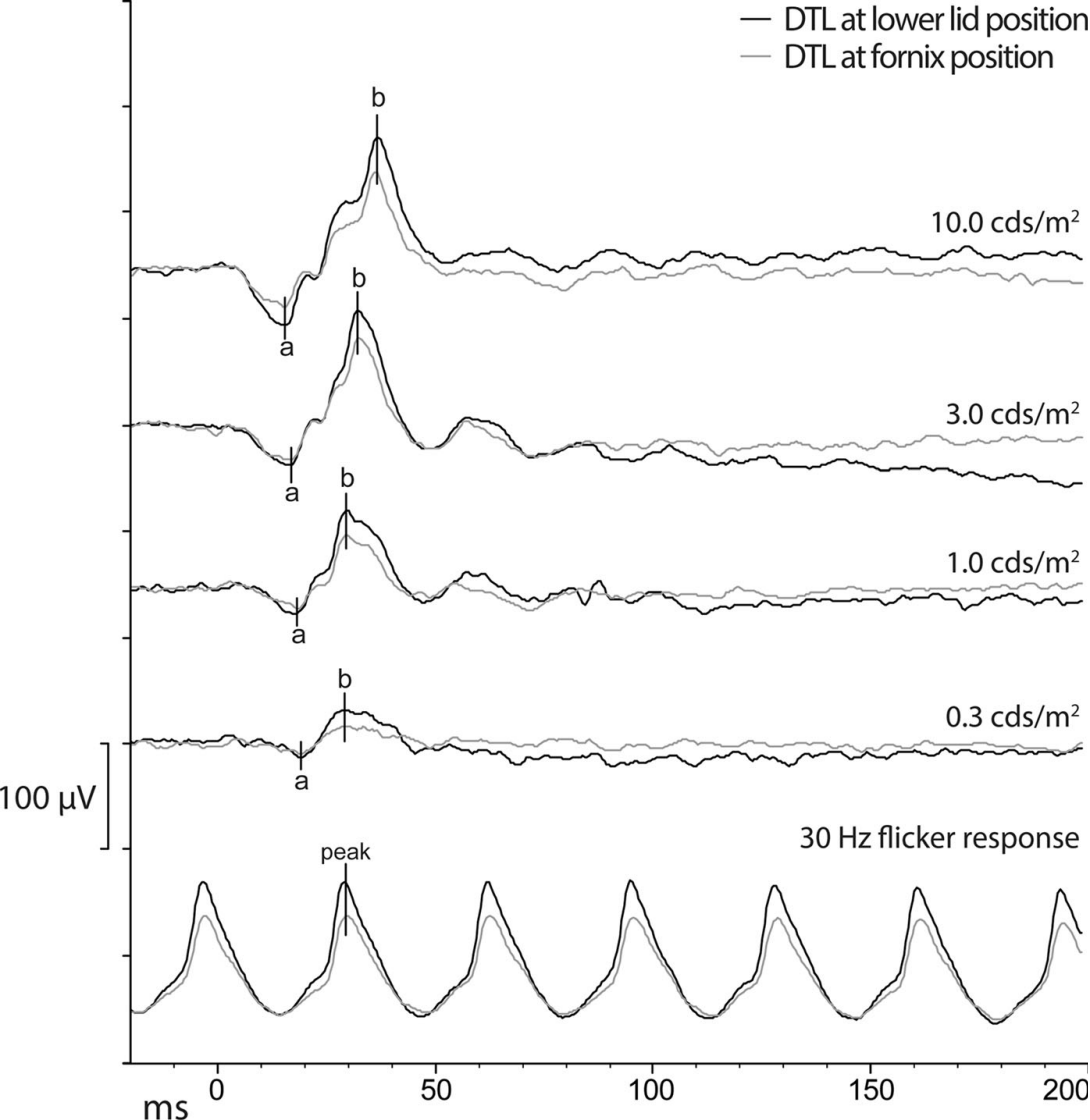
Example of ERG curves of the two DTL positions. Representative example of ERG curves obtained from the same patient with the DTL at the lower lid position (black) and the fornix position (gray). Abbreviations: DTL Dawson, Trick, and Litzkow electrode, ERG electroretinogram

Table 1 shows the results of the ERG responses of the different DTL positions. Median amplitudes were significantly higher (between 1.12 and 1.19 for the different stimulus strengths, see Table 2), and amplitude ranges were wider for all responses recorded with LLP compared to the FP. In contrast, there were no significant differences in implicit times between the two DTL positions. Figure 2 shows a representative example of ERGs curves of both DTL positions obtained from the same patient.

**Table 1:** ERG results and differences of two DTL electrode positions

	Lower lid		Fornix		*P* value
	Median	[IQR]	Median	[IQR]	
a-wave amplitude*					
0.3	− 11.8	[-15.5, − 8.1]	− 10.1	[− 13.0, − 7.4]	< 0.001***
1.0	− 19.4	[− 25.1, − 14.3]	− 16.3	[− 20.1, − 11.9]	< 0.001***
3.0	− 29.7	[− 37.8, − 22.2]	− 24.9	[− 30.5, − 19.8]	< 0.001***
10.0	− 46.1	[− 57.2, − 35.4]	− 37.4	[− 45.9, − 31.4]	< 0.001***
a-wave implicit time*					
0.3	19.0	[18.2, 20.0]	18.9	[18.0, 20.1]	0.445
1.0	17.1	[16.4, 18.0]	17.1	[16.3, 17.8]	0.115
3.0	15.7	[15.1, 16.4]	15.8	[15.1, 16.5]	0.816
10.0	14.8	[14.3, 15.5]	14.8	[14.2, 15.5]	0.119
b-wave amplitude*					
0.3	33.3	[23.3, 42.0]	26.2	[20.4, 34.6]	< 0.001***
1.0	76.0	[55.5, 97.6]	63.1	[49.9, 82.4]	< 0.001***
3.0	119.8	[92.2, 151.7]	103.2	[81.7, 129.1]	< 0.001***
10.0	118.4	[93.0, 144.6]	105.0	[82.6, 122.8]	< 0.001***
b-wave implicit time*					
0.3	27.0	[25.8, 30.0]	27.2	[25.6, 29.9]	0.718
1.0	28.9	[27.8, 31.0]	28.8	[27.7, 30.8]	0.105
3.0	31.6	[30.7, 33.0]	31.6	[30.7, 33.0]	0.129
10.0	35.9	[34.8, 37.3]	36.0	[34.7, 37.2]	0.055
30 Hz flicker peak					
Amplitude	67.1	[47.2, 86.3]	58.0	[43.9, 74.7]	< 0.001***
Implicit time	29.1	[27.5, 31.3]	29.1	[27.6, 31.3]	0.461

Descriptive statistics of the ERG responses of the two DTL positions (LLP and FP)

*Results of different flash strengths in candela. seconds/meters squared of the light-adapted ERG

***Statistically significant

*ERG* Electroretinogram, *DTL* Dawson, Trick, and Litzkow electrode, *IQR* interquartile range, *LLP* lower lid position, *FP* fornix position

**Table 2:** Amplitude ratio and implicit time difference between the two DTL positions

Amplitude ratio	Median	[IQR]	Percentiles	
			2.5th	97.5th
a-wave				
0.3*	1.18	[0.85, 1.56]	0.35	3.73
1.0*	1.18	[0.93, 1.52]	0.55	3.06
3.0	1.19	[0.98, 1.43]	0.55	2.34
10.0	1.19	[1.00, 1.44]	0.71	2.11
b-wave				
0.3*	1.19	[1.01, 1.39]	0.73	2.23
1.0*	1.13	[1.00, 1.33]	0.79	1.80
3.0	1.15	[1.00, 1.33]	0.78	1.75
10.0	1.12	[1.00, 1.31]	0.76	1.79
30 Hz flicker response				
	1.12	[0.97, 1.31]	0.73	1.80

Implicit time difference	Mean	(SD)	Percentiles	
			2.5th	97.5th
a-wave				
0.3*	0.03	(1.32)	− 3.22	2.47
1.0*	0.07	(0.92)	− 1.75	1.69
3.0	− 0.02	(0.79)	− 2.18	1.50
10.0	0.05	(0.72)	− 1.41	1.31
b-wave				
0.3*	− 0.03	(1.36)	− 2.85	2.93
1.0*	0.02	(0.85)	− 1.71	1.64
3.0	0.04	(0.80)	− 1.92	1.90
10.0	0.05	(0.63)	− 1.61	1.48
30 Hz flicker response				
	0.01	(0.85)	− 1.73	1.70

Results of the amplitude ratio (lower lid position/fornix position) and implicit time difference (lower lid position–fornix position) of the different flash strengths in candela. seconds/meters squared of the light-adapted ERG

*Candela·seconds/meters squared

To highlight what the effects may be of an unnoticed shift in DTL position on ERG results, we compared the results of the LLP and the FP to our reference values (obtained with the DTL in the LLP). Here, amplitudes were much more frequently defined as abnormal when the FP was used (supplementary Table 1). However, in 5% (20 eyes) the ERG was defined as abnormal in the LLP, but normal in the FP.

**Fig. 3:**
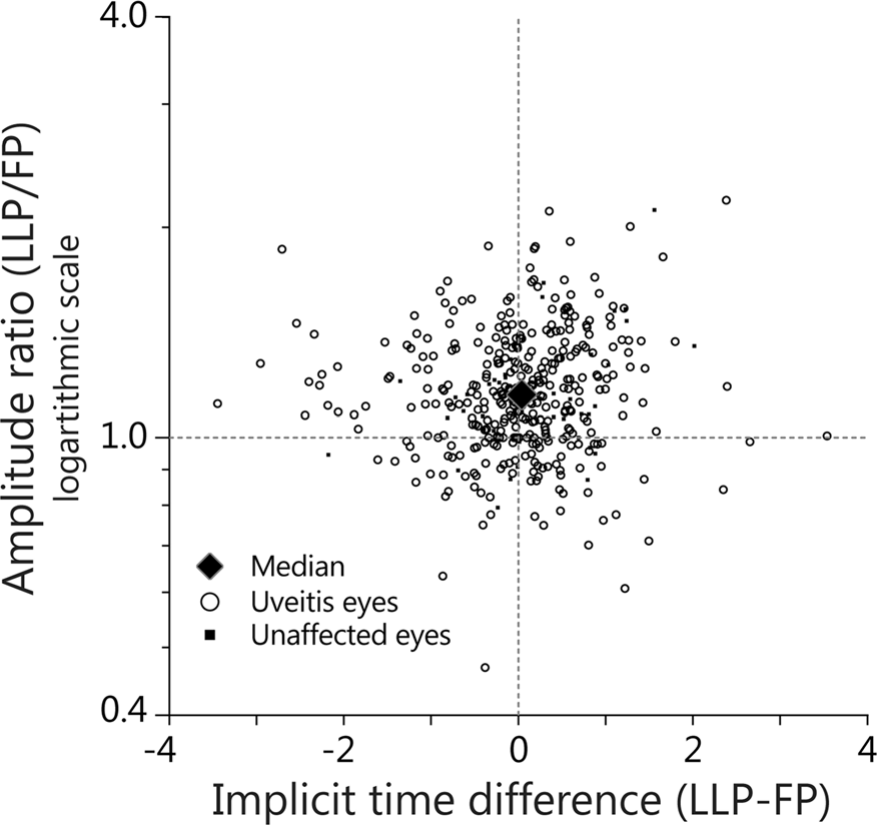
Amplitude ratio and implicit time differences of the two DTL positions. Scatter plots showing the implicit time differences (implicit time of LLP–LP) and amplitude ratio (LLP/FP) of the electroretinogram results all eyes for the light-adapted b-wave 3.0 a cds/m^2^. Uveitis eyes are indicated in as circles and unaffected eyes as squares. The large diamond indicates the median amplitude ratio (1.15) and mean implicit time difference (0.07). Abbreviations: DTL Dawson, Trick, and Litzkow electrode, cds/m^2 cd^ · seconds/squared meters, LLP lower lid position, FP fornix position

Figure 3 shows the amplitude ratio LLP/FP and the implicit time difference LLP–FP, for the b-wave (3.0 cds/m2) of both uveitis eyes (circle) and unaffected eyes (square). It illustrates that in most cases the amplitude ratio is higher than 1.0 which implies that the amplitude that was measured at the LLP was higher than at the FP. In contrast to this, the implicit time difference is evenly spread around zero, which implies that there was no tendency for the implicit time to be either higher or lower in one of the DTL positions.

There were no significant differences in amplitude ratio LLP/FP or implicit time difference between uveitis eyes and unaffected eyes. Supplementary Fig. 1 shows the same plots as Fig. 3 but for a-waves, other flash strengths of the b-wave and the 30 Hz flicker response. As is to be expected, the biggest variance between the two DTL positions is seen at lower stimulus strengths, because the signal is relatively low compared to the noise. The implicit time difference is evenly spread around zero in all measuring conditions. However, the amplitude ratio was in approximately 75% higher than 1.0. This implies that in approximately 75% of eyes the amplitude was higher in the LLP compared to the FP.

The ICC showed a good reliability between the two DTL locations for both the amplitude and the implicit time of the b-wave (ICC 0.842–0.973), with the ICC of the implicit time showing especially good reliability (Table 3). The ICC of the a-wave showed less good reliability. The ICCs of the weakest flash strengths were as low as 0.584. Bland–Altman plots show similar results (see supplementary Fig. 2).

**Table 3:** Intraclass correlation coefficient between the two DTL positions

	ICC	CI	
a-wave amplitude*			
0.3*	0.584	0.494	0.658
1.0	0.654	0.578	0.715
3.0	0.678	0.608	0.736
10.0	0.716	0.655	0.767
a-wave implicit time*			
0.3	0.788	0.742	0.826
1.0	0.830	0.793	0.860
3.0	0.856	0.825	0.882
10.0	0.857	0.826	0.883
b-wave amplitude*			
0.3	0.865	0.836	0.889
1.0	0.900	0.878	0.918
3.0	0.885	0.860	0.905
10.0	0.842	0.807	0.870
b-wave implicit time*			
0.3	0.946	0.934	0.955
1.0	0.973	0.967	0.978
3.0	0.954	0.944	0.962
10.0	0.982	0.978	0.985
30 Hz flicker Peak			
Amplitude	0.897	0.874	0.915
Implicit time	0.979	0.975	0.983

ICCs showing reliability between the ERG with two DTL positions: lower lid position and fornix position

*Results of different flash strengths in candela seconds/meters squared of the light-adapted ERG

*ICC* Intraclass correlation coefficient, *ERG* electroretinogram, *DTL* Dawson, Trick, and Litzkow electrode, *CI* confidence interval

Regarding the amount of variance, amplitude ranges of both positions were quite large, but they were significantly larger in the LLP compared to the FP. However, the CoV did not differ between the two DTL positions, implying that the relative variability was not statistically significantly different between the two positions (Table 4).

**Table 4:** Coefficients of variation of amplitudes of the two DTL positions

	Lower lid position	Fornix position	*P* value
a-wave*			
0.3	24.2	21.8	0.054
1.0	19.6	19.3	0.904
3.0	17.7	15.7	0.014
10.0	15.5	14.5	0.202
b-wave*			
0.3	21.2	19.7	0.187
1.0	20.5	19.3	0.267
3.0	36.0	33.7	0.239
10.0	32.7	30.8	0.259
30 Hz flicker*			
	21.8	20.5	0.256

CoV of amplitudes were calculated after data were transformed to fit a normal distribution of two DTL positions (lower lid position and fornix position). The R package cvequality (version 0.1.3; Marwick and Krishnamoorthy 2018) was used to test for significant differences in CoV of the two DTL positions. Based on Bonferroni’s correction (more tests than shown in this table), *P* values below < 0.006 were considered as statistically significant

*Results of different flash strengths in candela seconds/meters squared of the light-adapted ERG

*ERG* Electroretinogram, *DTL* Dawson, Trick, and Litzkow electrode, *CI* confidence interval, *CoV* coefficients of variation

## Discussion

The aim of this study was to investigate whether one of two commonly used DTL positions is superior for monitoring purposes in patients. We found no differences in implicit times. We did find differences in amplitudes: In the LLP the amplitudes were between 1.12 and 1.19 times higher compared to the FP and the ranges were larger as well. These differences are lower but still comparable to other reports, where mean amplitudes in the LLP were between 20 and 31% higher compared to the FP [10, 12, 19]. However, these previous reports did not investigate whether there was a statistically significant difference in relative variability. Some investigate whether the variability, or amount of agreement (ICC), differs between the two positions [10]. While others report that the variance decreases by 20% in the FP. If the relative variability, or relative standard deviation decreases as well, is not investigated [12]. This study shows that the relative variability was not statistically significantly different between the two DTL positions.

It is difficult to compare our results to the literature, because most studies do not investigate DTL position but investigate the DTL electrode to another type of electrode. Also, some reports investigate DTL position in different test such as the multifocal ERG [10] or pattern ERG [19]. Kurtenbach et al. did investigate the effects of DTL position on the full-field ERG. However, they investigated a smaller group (13 subjects) and did not measure the ERG on the same day. The differences they found were slightly larger than in our study, with a mean difference in amplitude of 20% (SD 9.71%) for the b-wave and 27% (SD 17.7%) for the a-wave of the LA 3.0 cds/m^2^. The smaller sample size and the relatively large SD could explain the differences with our study.

Also, they did not investigate differences in the relative variability. We found the differences in amplitude between the LLP and FP to be proportional, because the higher amplitude in the LLP is accompanied by a larger range and the lower amplitude in the FP is accompanied by a smaller range. Therefore, we cannot conclude that one position yields more stable results than the other and should be used for monitoring purposes. Thus, we think other factors should be taken into account when a DTL position is chosen for local protocols.

If patient comfort is considered most important, the FP may be preferred [10]. Another advantage of the FP is that the chance of scratching the cornea may be lower than in the LLP. The downside of the FP is that responses might become too small to detect at all. Therefore, the LLP may be preferred for monitoring diseases such as retinitis pigmentosa, where it is essential to be able to detect even the smallest ERG responses.

Whichever position is chosen, it is crucial to consistently use the same DTL position. This is both important to determine whether an ERG is abnormal and important to determine whether an ERG has improved or worsened.

If the DTL position has shifted from one position to the other during an ERG measurement, this must be taken into account when the ERG is reviewed. It is possible to correct for the shift by multiplying the amplitude response with the corresponding median amplitude ratio. However, clinicians must be aware of the quite large percentile ranges of the amplitude ratios. Also, in some eyes amplitudes were higher in the FP compared to the LLP, which might be due to shifts of the DTL in an upward direction. Why amplitudes recorded at the FP are lower than in the LLP, remains speculative. But, it can probably be attributed to differences in recording resistance, which is lower at the cornea, and higher along the sclera and especially the ora serrata [17, 20].

Despite the large amplitude range, the reliability was good for both amplitudes and implicit times of the b-wave and the 30 Hz flicker response. Regarding amplitudes, this implies that the measurements have consistent results, although the response is generally lower in the FP than in the LLP.

The reliability for the a-wave amplitude was worse, especially in the dimmer flashes. This could be explained by small trends in the ERG response. These trends alter the slope ERG response in a linear fashion. Ideally these trends are corrected by “toggling” the response, thereby removing the added slope. However, this was not always possible due to blinks at the end of the ERG response. Such unaltered small trends may have a relative large effect on small responses. Also, trends generally affect the a-wave more, since the a-wave amplitude is an absolute measure relative to zero, while the b-wave amplitude is a measure relative to the a-wave.

Our study population consisted of (uveitis) patients, instead of healthy volunteers. When patients are measured, multiple factors may influence the results: Patients might be more anxious, be photophobic, have a higher sensibility of the eye, or be frightened of the procedure in general. Therefore, the results of this study may reflect the conditions of clinical practice more accurately than a study with healthy volunteers who are frequently familiar with the proceedings.

However, it is important to note that uveitis patients have a great intra-individual variability, ranging from normal to almost absent responses [18]. This explains why the ranges from this study are relatively large and often not normally distributed. That being said, we believe that the conclusions drawn from this study can be extrapolated to other populations, because we tested both DTL positions in the same patient and compared these with each other.

A limitation of this study is that the LLP was always measured first and the FP second. Alternating between these two positions at random would have been preferable to correct for a possible unknown bias. Since our reference values were measured with the LLP, we wanted to make sure we first obtained an ERG which was compliant with our reference values, before measuring other ERG results.

Also, in future studies it would be interesting to measure the ERG twice in the FP and twice in the LLP. This would give an even better indication of the amount of variability between the two positions.

In summary, we conclude that neither DTL position is preferred above the other when monitoring patients. When clinicians choose a DTL position for new reference values, they must decide whether they prefer larger responses or greater patient comfort. But above all, it is important to check the DTL position during an ERG measurement and to ensure that the DTL position is the same as in earlier measurements of the same patient.

## Electronic supplementary material

Below is the link to the electronic supplementary material.

Supplementary material 1 (TIFF 71654 kb)

Supplementary material 2 (TIFF 75417 kb)

Supplementary material 3 (DOCX 20 kb)
