# Supplementary material for: Effects of DTL electrode position on the amplitude and implicit time of the electroretinogram
Source: Doc Ophthalmol. 2019 Nov 4;140(3):201–9. doi: 10.1007/s10633-019-09733-3 (PMC7205847; doi:10.1007/s10633-019-09733-3)
Supplement: Supplementary file 3 — Supplementary material 3 (DOCX 20 kb) [file 10633_2019_9733_MOESM3_ESM.docx]

**Supplementary material**

**Effects of DTL electrode position on the amplitude and implicit time of the electroretinogram.**

Documenta Ophthalmologica

Anna.H. Brouwer^1,2^, Gerard.C. de Wit^1^, Joke.H. de Boer^2^, Maria.M. van Genderen^1,2^

Affiliations:

1. Bartiméus Diagnostic Centre for complex visual disorders, Zeist, The Netherlands

2. Department of Ophthalmology, University Medical Centre Utrecht, Utrecht, the Netherlands

Contact information corresponding authort:

Email: [A.H.Brouwer-7@umcutrecht.nl](mailto:A.H.Brouwer-7@umcutrecht.nl)

Telephone: +31631969442

Adress: Heidelberglaan 100

3584 CX Utrecht, the Netherlands

**Supplementary table 1: Differences in amount of ERG abnormalities of the two DTL positions.**

|  | | **Lower lid position** | | **Fornix position** | | **P-value** |
| --- | --- | --- | --- | --- | --- | --- |
|  |  | N | % | N | % |  |
| Single flash cone | |  |  |  |  |  |
| A-wave | Amplitude | 54 | (13.5) | 96 | (24.0) | <0.001* |
|  | Implicit time | 123 | (30.8) | 115 | (28.7) | 0.312 |
| B-wave | Amplitude | 95 | (23.8) | 146 | (36.5) | <0.001* |
|  | Implicit time | 154 | (38.5) | 150 | (37.5) | 0.522 |
| 30 Hz flicker response | |  |  |  |  |  |
|  | Amplitude | 128 | (32.0) | 155 | (38.8) | 0.001* |
|  | Implicit time | 125 | (31.2) | 126 | (31.6) | <0.999 |

Number of ERG abnormalities of the two DTL positions (LLP and FP). An ERG was considered abnormal if amplitudes were below the 5th percentile, or implicit times were above the 95th percentile, or both, when compared to our reference values. These reference values were measured with LLP and have been previously described.^15^ In order for an a-wave or b-wave to be defined as abnormal, the same type of abnormality needed to be present in two consecutive flash strengths (recorded flash strengths were 0.3, 1.0, 3.0 and 10.0 candela · seconds/squared meters)

* statistically significant.

Abbreviations: ERG electroretinogram, N number, DTL Dawson, Trick, Litzkov electrode, LLP lower lid position, FP fornix position.

**Supplementary figure 1: Amplitude ratio’s and implicit time differences of the two DTL positions.**

Scatterplots showing the implicit time differences (LLP – FP) and amplitude ratio (LLP / FP) of the electroretinogram results all eyes of the different flash intensities for the a-wave (0.3, 1.0, 3.0, 10.0 a cds/m^2^)., b-wave (0.3.1.0, 10.0; for 3.0 see figure 2) and the peak of the 30Hz flicker response (3.0 a cds/m^2^). Uveitis eyes are indicated in as circles and unaffected eyes as squares. The large diamond indicates the median amplitude ratio and mean implicit time difference. Abbreviations: DTL Dawson, Trick, Litzkov electrode cds/m^2^ candela · seconds/squared meters, lower lid position LLP, fornix position FP.

**Supplementary figure 2: Bland Altman plots of the two DTL positions.**

Bland Altman plots of the implicit time differences (LLP- FP) and amplitude ratio (LLP/FP) of the electroretinogram results all eyes of the different flash intensities for the a-wave (0.3, 1.0, 3.0, 10.0 a cds/m^2^)., b-wave (0.3.1.0, 3.0, 10.0) )and 30Hz flicker response (3.0 a cds/m^2^).

Abbreviations: DTL Dawson, Trick, Litzkov electrode cds/m^2^ candela · seconds/squared meters, lower lid position LLP, fornix position FP.
